# Visual Disturbances Spectrum in Pediatric Migraine

**DOI:** 10.3390/jcm12082780

**Published:** 2023-04-08

**Authors:** Ilaria Frattale, Laura Papetti, Fabiana Ursitti, Giorgia Sforza, Gabriele Monte, Alessandra Voci, Martina Proietti Checchi, Luigi Mazzone, Massimiliano Valeriani

**Affiliations:** 1 Child Neurology and Psychiatry Unit, Systems Medicine Department, Hospital of Rome, Tor Vergata University, 00165 Rome, Italy; 2 Developmental Neurology, Bambino Gesù Children Hospital, IRCCS, 00165 Rome, Italy; 3 Center for Sensory Motor Interaction, Aalborg University, 9220 Aalborg, Denmark

**Keywords:** migraine, pediatric, childhood, alice in wonderland, visual snow syndrome, pathophysiology

## Abstract

Migraine is a complex neurological disorder with partially unknown pathophysiological mechanisms. The prevalence in childhood ranges from 7.7% to 17.8%, thus representing the most frequent primary headache. In half of the cases, migraine is accompanied or preceded by various neurological disturbances, among which the visual aura is the best known. In literature, other conditions, such as Alice in Wonderland Syndrome and Visual Snow syndrome, are characterized by visual manifestations and are often associated with migraine. The aim of this narrative review is to describe the spectrum of visual disturbances in pediatric migraine and their pathophysiological mechanisms.

## 1. Introduction

Pediatric migraine is a frequent neurological disorder that affects children and adolescents in a percentage that increases with age from 7.7% to 17.8%, with female sex predominance starting from puberty [1,2,3,4]. Migraine can be preceded or accompanied by transient neurological symptoms termed aura, which affects 1.6% of pediatric migraineurs. In two-thirds of cases, aura’s main symptom is visual [5]. Visual aura presents a heterogeneous manifestation and has been largely investigated both in adults and children [6,7,8,9,10].

In 1952 Caro Wolfram Lippman described seven cases with complex symptomatologies that, 3 years later, were defined as Alice in Wonderland Syndrome (AIWS) in honor of Lewis Carroll’s character by the psychiatrist John Todd [11]. AIWS is characterized by transient visual hallucinations and temporospatial dysperceptions, with derealization preceding a migraine attack.

Visual snow syndrome is characterized by the continuous and unremitting vision of dots over the entire visual field, similar to an incorrectly programmed analog TV [12]. In 40% of cases, this disorder is present from childhood [13].

The aim of our review is to describe the spectrum of visual disturbances associated with pediatric migraine and to underline how they share common pathophysiologic mechanisms with migraine.

## 2. Clinical Manifestations

### 2.1. Migraine with Visual Aura

Criteria used to diagnose migraine with aura are defined by the International Headache Society (IHS). They include the presence of at least one symptom among visual, sensory, speech/language, brainstem, motor and retinal, lasting from 5 to 60 min. If more than one symptom is present, they must occur in succession, and at least one symptom must be positive and unilateral [14].

Aura symptoms are visual in almost all cases [15,16], while sensory and language symptoms involve about 30% and 10% of patients, respectively [10]. Studies have demonstrated that patients with visual aura have a lower frequency and shorter duration of headache attacks compared to patients with multiple symptoms [8,15,17,18].

Visual aura is characterized by a variety of manifestations, which were classified by Viana et al. on the basis of a literature review [19]. They proposed 25 names to classify aura manifestations: 1. bright light; 2. foggy/blurred vision; 3. zigzag lines; 4. scotoma for single-blind areas; 5. scotomata for several blind/black areas; 6. small bright dots; 7. white dots/round forms; 8. colored dots/round forms; 9. lines (colored); 10. geometrical shapes; 11. like looking through heat waves, water or oil; 12. visual snow; 13. bean-like forms; 14. hemianopsia; 15. deformed images for alterations in lines/angles; 16. tunnel vision for blindness in the whole periphery; 17. oscillopsia for movement of stationary objects; 18. mosaic vision; 19. fractured objects; 20. corona effect for an extra edge of an object; 21. anopia for total blindness; 22. micropsia; 23. macropsia; 24. like a negative film; 25. complex hallucinations for visual perception of something not present.

A prospective study showed that more than half of 72 patients suffering from migraine with aura had not reported a stereotyped aura during three consecutive attacks. In two-thirds of the cases, more than one visual symptom was described [17].

Blurred vision is the most frequent visual manifestation of migraine [20], ranging from 25% to 54% of patients, along with other aura symptoms [10,21,22]. It often lasts hours, exceeding the duration of classic aura. Despite its high prevalence, this symptom cannot be considered a visual aura since it has a peripheral origin (i.e., pupillary dysfunction), while the aura is attributed to an activation of the brain or retina [20].

In childhood, an aura is present in about 1.6% of cases [23]. Balestri et al. evaluated whether the diagnostic criteria of ICHD-3 are more sensitive than those of ICHD-2 to diagnose migraine with aura in childhood. Through the analysis of clinical characteristics (i.e., location and quality of pain, duration of the attack, type and duration of the aura) of 164 children suffering from migraine with aura, it was possible to diagnose migraine with typical aura in 77.1% of cases using the ICHD-3 criteria and 15.3% with the ICHD-2 criteria, demonstrating that the new classification can be used confidently also for pediatric age [8]. However, in childhood, there may be unusual clinical features that make diagnosis challenging. In adults, symptoms are generally unilateral, while in pediatric age are bilateral in more than 50% of cases, with a duration of visual aura that is shorter than 5 min in about 20% of cases [8,24]. Despite IHS consensus on aura’s duration criterion and its possible application also in pediatric age, in clinical practice, a non-negligible percentage of pediatric patients have an aura lasting less than 5 min. While using the IHS diagnostic criteria, we should consider that a consensus is not scientific proof and may not cover 100% of cases.

Usually, migraine aura lasts between 5 and 60 min with colorless images and is unilateral, sparkling with gradual onset and termination, followed by headache after several minutes. Many authors [15,21,22,25,26,27,28,29] reported that aura could be shorter and with colored images. Although these symptoms may suggest Gastault’s occipital epilepsy, it must be emphasized that epileptic visual hallucinations are different. Specifically, they develop in a few seconds and last 1–3 min, with colored images showing circular patterns that start peripherally in the temporal side of the visual hemifield and spread to the other side. These images have a stereotyped character in each patient and are associated with other signs, such as eye and head deviation [30,31]. Ictal blindness, present in 65% of epileptic patients, is sudden and lasts 3–5 min [31,32,33,34]. In one-third of cases, there is a postictal headache that is difficult to distinguish from a migraine attack [31,32,33,35]. Epilepsy and migraine have a close, albeit debated, link [36]. A retrospective study involving 1795 children with headaches showed that in the migraine population, the risk of epilepsy is 3.2 times greater than in those suffering from tension-type headaches, while epileptic children have a 4.5 times greater risk of developing migraine than tension-type headaches [37]. From a pathophysiological point of view, epilepsy and migraine share ionic dysfunction and cortical hyperexcitability [38]. In support of this association, hemicrania epileptica, postictal headache and migraine aura-triggered seizure, known as migralepsy, are described in ICHD3. Migralepsy is a condition in which, during or within 1 h from the onset of an aura attack, an epileptic seizure occurs in a person with a history of migraine with aura. As described in the literature, most patients with migralepsy occipital epilepsy cannot be excluded [39,40], so they should be diagnosed more correctly with “migraine aura-triggered seizures”, as proposed by Sforza et al. [41].

### 2.2. Typical Aura without Headache

As mentioned in ICHD3, the aura precedes or accompanies the headache, and it can even present at the end of the headache attack. However, some patients may have an aura not followed or accompanied by a headache, called typical aura without headache (TAH), previously known as acephalic headache [14].

TAH occurs throughout the patient’s life, with a higher prevalence in the female population [15,42], with a peak between 20 and 39 years and between 60 and 69 years [43]. It occurs in both adults and children [8]. In 25% of cases, it is characterized by visual aura alone [44], but symptoms of different modalities can occur in the same episode. In almost all cases, visual symptoms are positive (colored images or bright lights) [44,45,46,47]. In the literature, there are descriptions of patients with aura alone without evidence of headache [45,46] or with just a mild headache and episodes of aura without headache [48]. Some cases of retinal aura without headache in subjects with a history of migraine with retinal aura [49] are reported in the literature. Manifestations are often one-sided, transient and mostly with only negative symptoms [50]. Differential diagnosis between transient ischaemic attack, central retinal artery occlusion, emboli, retinal vein occlusion, or ischemic optic neuropathy can be difficult [51,52].

### 2.3. Alice in Wonderland Syndrome

AIWS is a rare condition that can be associated with neuropsychiatric manifestations, migraine, intoxications, tumors, epilepsy [53], as well as feverish states and infections with various pathogens, including Epstein Barr virus, cytomegalovirus, varicella, coxsackie B1 [54,55].

Subjects with AIWS, especially children, are reluctant to tell about their symptoms since they fear being labeled as patients with psychiatric disorders [53]. Therefore, this disturbance is possibly underestimated.

The core symptom of AIWS is body schema distortions associated with other symptoms [11,56,57]. Body dysperceptions are defined as microsomatoagnosia in the case of the perception that one’s body is shrinking or macrosomatoagnosia if there is the perception that one’s body is enlarging or distorting. Macrosomatoagnosia most frequently affects the head and upper body, resembling the disproportionate representation of the human body (homunculus) in the sensorimotor cortex [58,59]. Metamorphopsias or Lilliputian visual hallucinations, so named in honor of the imaginary island of Liliput described in the book “Gulliver’s Travels” by J. Swift in 1726, include micropsia or macropsia, depending on whether objects appear smaller or larger, teleopsia if objects appear distant, pelopsia if objects appear close, and zoopsia if visions of animals occur. There may also be alterations in the perception of time (slow-fast) and the distortion of sounds that can either be perceived as distorted or amplified [53,60]. Lastly, visual or auditory hallucinations and dyschromatopsia can be part of the AIWS. All symptoms are accompanied by a dream-like state [11], derealization and depersonalization. Todd also defined hyperschematia as a condition of left-sided distortion with the perception of the disproportionate expansion of space towards the left [61]. Memory loss, emotional instability, anxiety, abnormal movements of limb, persistence of hearing sounds have been also described [60].

A correlation between AIWS and migraine, consisting of a personal or family history of migraine, was noticed from earlier observations, and Lippman also suggested a close temporal relationship [58].

Patients with AIWS can be divided into three subgroups: type A with somesthetic symptoms (about 9%), type B with only visual symptoms (more than 75%), and type C with both visual and somesthetic symptoms (16%) [56]. Liu et al. [62] proposed to define type A as AIWS, while patients of B and C groups would be affected by Alice In Wonderland Like Syndrome (AIWLS).

Since AIWS is a typical condition of childhood and adolescence [56] and correlates with migraine, it can be considered as a migraine equivalent. In a prospective study about migraine patients followed up for 30 years, it was observed that distortions of space and time perception are reported for the first time even during adulthood [63], while in pediatric age most frequently reported symptoms are micropsia and teleopsia [62].

### 2.4. Visual Snow Syndrome

Visual Snow Syndrome (VSS) was first described by Liu et al. in 1995 [64] and consists of the persistent vision of positive visual phenomena (dots) over the entire visual field, with the perception of seeing an analog TV to be programmed. It should last at least 3 months and could persist for some years [13]. Symptoms of VSS can be misinterpreted as persistent visual auras.

Nyctalopia, entopic phenomena, palinopsia, and photophobia are often associated with VSS. Nyctalopia is defined as disturbed night vision. Palinopsia is the persistence of seeing images without being able to suppress them. Entopic phenomena are defined as at least one of the following manifestations: spontaneous photopsia (i.e., bright flashes), self-light of the eyes (i.e., the vision of round colored images when the eyes are closed in the dark) and the entopic phenomenon of the blue field looking at a bright surface.

The dots are typically white or gray on a black background or gray and black on a white background, although cases of glittering, colored or transparent dots have been described [14]. VSS diagnostic criteria are shown in Table 1 [14].

The disturbance must not be caused by the ingestion of hallucinogenic drugs, and the evaluation of visual function is normal. Symptoms are visible both with eyes open and closed.

There are only a few case reports of VSS in children, exclusively in association with migraine [65,66,67,68], and it has only recently been investigated in adulthood. In 2020, Puledda et al. evaluated the clinical characteristics and comorbidities of more than 1000 adult patients with VSS. Onset in childhood was reported in 40% of cases, with the persistence of symptoms lifelong; in one-third of the cases, symptoms started during a migraine attack, and 75% of the patients had tinnitus as a comorbidity [13].

## 3. Pathophysiology

In the 1940s, animal model studies [69,70,71,72] suggested that the mechanism underlying migraine aura is cortical spreading depression (CSD), a depolarization wave, following cortical hyperactivation, which proceeds from the posterior to anterior brain region with initial involvement of visual cortex [69]. However, this has never been demonstrated in humans, and the link between aura and CSD is only hypothetical. The depolarization wave had a speed of 3 mm/s, and the progressive cortical involvement does not explain an aura duration of 180 min [73]. Some authors hypothesized that the simultaneous presence of different aura symptoms could be related to distinct mechanisms, with the thalamus playing a key role [74,75]. According to Martens-Mantai [76] et al., the contribution of other areas, such as the entorhinal cortex, should be considered. Olesen et al. studied visual aura using the intra-arterial infusion of Xe-133 and described initial hyperemia followed by a reduced blood flow starting from the occipital toward the anterior regions, not attributable to ischemia. These data suggested that migraine aura is both a neuronal and vascular phenomenon [77,78], with an alteration that begins in the central visual field and goes peripherally [79]. To study the progression of visual aura, functional magnetic resonance studies used the BOLD (Blood-oxygen-level-dependent) technique during visual stimuli. Progression of visual aura symptoms was related to an increase and subsequent reduction of the BOLD signal, which starts from the extrastriate area V3A and proceeds to the visual cortex [79,80,81] (Table 2). It has also been observed that positive or negative aura symptoms correlate with an altered BOLD in the visual cortex [82]. The heterogeneity of visual aura is not explained by distinct pathophysiologic mechanisms, and it could be caused by diverse CSD behaviors or different supplementary visual cortex involvements [83].

Various hypotheses have been made on the pathophysiology of AIWS, such as cortical dysfunction due to cerebral hypoperfusion, encephalitis, or epileptic activity [84,85]. The core area of AIWS seems to be the TPO-C (temporo-parietal-occipital carrefour) [86], which is found between the parietooccipital, temporooccipital, and temporoparietal cortices where visual, somatosensory and vestibular information is integrated. The key areas of the TPO-C are the angular gyrus, which is activated in all patients who experience complex sensory disturbances, and the POJ (parieto-occipital junction), which is involved in unconventional spatial cognition (Table 2) [87,88]. Body distortion is possibly due to stimulation of the posterior parietal cortex [89] or hypoperfusion of the posterior non-dominant parietal lobe [86]. Using SPECT [90] and other nuclear medicine techniques, studies reported hypoperfusion of the temporo-occipital and perisylvian regions, frontoparietal and frontal areas [89] during micropsia, while hypoperfusion of the frontotemporal operculum corresponded to macro- and microsomatoagnosia and teleopsia [91]. A recent resting-state functional connectivity study demonstrated a similar connectivity alteration in AIWS and migraine with aura compared to healthy controls. In particular, functional connectivity is decreased in medial and lateral visual network, in the areas considered the “aura generator” (i.e., superior lateral occipital cortices and the lingual gyrus) [50] while it is increased in salience (i.e., right insula) and default mode network (i.e., the posterior cingulate cortex). Comparing AIWS to MA, greater alterations in functional connectivity was found in AIWS, especially in executive control network regions and basal ganglia [92].

The pathophysiology of VSS is largely unknown, but hypotheses have been proposed. Considering that the entire visual field is affected, the alteration of visual pathways as the primary visual cortex or optical radiation can be excluded. The symptoms of VSS (i.e., palinopsia and entopic phenomena) are related to the processing of visual information, so the supplementary visual cortex is involved. This is demonstrated by a PET study that showed a hyper-uptake of fluorodeoxyglucose of lingual gyrus, part of the supplementary visual cortex [93], and by a voxel morphometry study showing grey matter thickness of the same area [94,95]. Broadman area 19, corresponding to the extrastriate visual association cortex, is linked to other extrastriate areas, including V3A, that are involved in migraine aura (Table 2) [79,96]. There are conflicting data regarding the hyperexcitability of the visual cortex [97], which is probably due to a fallacious inhibitory mechanism [98]. This is supported by delayed P145 response in visually evoked potentials [99] and a higher visual threshold in object discrimination [100,101], features also found in migraine patients [102,103,104]. Functional magnetic resonance studies demonstrated altered connectivity between visual, salience network, visual motion and attentional network areas [105]. Furthermore, reduced activity of the anterior insula after external visual stimuli may suggest the involvement of the salience network, which has the role of selecting the most relevant stimuli [106]. McKendrick [100] hypothesized an abnormal communication in the thalamocortical networks leading to poor visual control attention with more rapid eye movements towards new stimuli [99].

## 4. Association with Migraine

### 4.1. Alice in Wonderland Syndrome

Illusions and hallucinations may be present during a migraine attack and are difficult to distinguish in childhood [107]. If there is a migraine history, a positive family history of migraine and an absence of psychotic symptoms, they can be considered as part of the migraine aura attributable to AIWS [63,108,109]. Although AIWS is not classified in ICHD3, its correlation with migraine is very strong, as migraine is the second cause (26.8%) of AIWS in children [88]. The association of AIWS with vestibular migraine attacks is reported in 77% of patients with vestibular migraine [110]. Moreover, abdominal colic pain, which is considered a migraine equivalent, can occur during AIWS [111].

The following criteria have been proposed by Valença et al. [112] for diagnosis of migraine-related AIWS: a. one or more episodes of metamorphopsia or self-experienced body schema illusion; b. duration less than 30 min; c. history of migraine or followed by migraine; d. normal neuroimaging, EEG, and cerebrospinal fluid examination.

From the pathophysiological point of view, the association between migraine and AIWS suggests an involvement of electrical activity with CSD that determines a wave of depolarization in the cortical neurons of the TPO-C areas [113,114]. The highest frequency in children is explained by the immaturity of myelination of the associated cortical structures, which caused a greater vulnerability to CSD compared to adults [115].

### 4.2. Visual Snow Syndrome

Since VSS’s initial description, its correlation with migraine has become more solid, and this condition is currently included in the appendix of the ICHD3 as a complication of migraine [14]. It has been demonstrated that after 6 years from the onset, 94% of patients keep meeting the criteria for VSS, and 47% meet the diagnostic criteria for migraine [116]. Another study has shown that 59% of VSS patients also have migraines, and 27% show a typical aura, suggesting common pathophysiologic mechanisms [12].

As mentioned above, in VSS, there is an involvement of the lingual gyrus and V3A area, parts of the supplementary visual cortex (Broadmann area 19), which are thought to be linked to photophobia, a symptom shared by VSS and migraine [117]. The link between migraine and VSS could be the cortical microstructure of area 19 that extends from the lingual gyrus to other areas, including area V3A, which undergoes the initial functional changes during the migraine aura [79].

## 5. Treatment

Animal studies showed that antiepileptic drugs, such as carbamazepine [118], phenytoin [119], and ketamine [120], block CSD in animals, but the same medications do not have any positive effect on migraine prevention in humans [121,122]. Lamotrigine is effective in the prophylaxis of migraine with aura [123,124,125,126] but not in migraine without aura [125,127].

In the case of AIWS, the associated diseases (i.e., migraine, epilepsy, infections) should be treated [61].

To date, no trials for the treatment of VSS have been reported. Some studies have demonstrated the effectiveness of therapies used for migraines, such as lamotrigine, antidepressants [128], and verapamil [129]. Retrospective data on 58 patients with VSS showed partial improvement with lamotrigine as compared to flunarizine, acetazolamide and valproate [130]. A prospective questionnaire on 40 VSS patients showed negative results with benzodiazepines, antiepileptics, antidepressants, and antibiotics [131]. Improvements with naproxen for acute attacks are reported [12].

## 6. Conclusions

Visual symptoms associated with migraine, though very common, still represent a pathophysiologic enigma, as suggested by the presence of multiple hypotheses about their etiology. Beyond the CSD, other mechanisms are likely to contribute to atypical aura symptoms and visual disturbances observed in VSS and AIWS. Several elements support the hypothesis of an association between migraine, VSS, and AIWS; thus, the most promising way to improve our knowledge is to investigate the functional changes of the brain in these conditions. In particular, it will be important to define the pathophysiologic mechanisms shared by these diseases, but also the peculiarities of each condition responsible for the different phenotypes.

## Figures and Tables

**Table 1:** VSS diagnostic criteria.

Diagnostic Criteria
A. Dynamic, continuous, tiny dots across the entire visual field,1 persisting for >3 months
B. Additional visual symptoms of at least two of the following four types: 1. palinopsia 2. enhanced entoptic phenomena 3. photophobia 4. impaired night vision (nyctalopia)
C. Symptoms are not consistent with typical migraine visual aura
D. Symptoms are not better accounted for by another disorder

**Table 2:** Putative pathophysiological pathways of migraine with aura, AIWS, and VSS.

	Pathophysiological Pattern
Migraine	CSD proceeds in a posteroanterior way in all directions starting from the visual cortex; Increase and subsequent reduction of the BOLD signal from the V3A area to the visual cortex
Alice In Wonderland Syndrome	CSD involves neurons of the TPO-C area Cortical dysfunction due to hypoperfusion
Visual Snow Syndrome	Hyperactivity of supplementary visual cortex V3A

## Data Availability

Not applicable.
